# A Systematic Review of Web-Based Interventions for Patient Empowerment and Physical Activity in Chronic Diseases: Relevance for Cancer Survivors

**DOI:** 10.2196/jmir.2281

**Published:** 2013-02-20

**Authors:** Wilma Kuijpers, Wim G Groen, Neil K Aaronson, Wim H van Harten

**Affiliations:** ^1^Netherlands Cancer InstituteDivision of Psychosocial Research and EpidemiologyAmsterdamNetherlands; ^2^University of TwenteDepartment of Health Technology and Services ResearchEnschedeNetherlands

**Keywords:** systematic review, Internet intervention, chronic disease, patient empowerment, physical activity, cancer survivor

## Abstract

**Background:**

Patient empowerment reflects the ability of patients to positively influence their health and health behavior such as physical activity. While interactive Web-based interventions are increasingly used in various chronic disease settings to enhance empowerment and physical activity, such interventions are still uncommon for cancer survivors.

**Objective:**

The objective of this study was to systematically review the literature regarding interactive Web-based interventions. We focused on interventions aimed at increasing patient empowerment and physical activity for various chronic conditions, and explored their possible relevance for cancer survivors.

**Methods:**

Searches were performed in PubMed, Embase, and Scopus to identify peer-reviewed papers reporting on randomized controlled trials that studied the effects of Web-based interventions. These interventions were developed for adults with diabetes, cardiovascular disease, chronic obstructive pulmonary disease, heart failure, or cancer. Intervention characteristics, effects on patient empowerment and physical activity, information on barriers to and facilitators of intervention use, users’ experiences, and methodological quality were assessed. Results were summarized in a qualitative way. We used the recommendations of the Institute of Medicine (IOM) regarding cancer survivorship care to explore the relevance of the interventions for cancer survivors.

**Results:**

We included 19 papers reporting on trials with 18 unique studies. Significant, positive effects on patient empowerment were reported by 4 studies and 2 studies reported positive effects on physical activity. The remaining studies yielded mixed results or no significant group differences in these outcomes (ie, no change or improvement for all groups). Although the content, duration, and frequency of interventions varied considerably across studies, commonly used elements included education, self-monitoring, feedback/tailored information, self-management training, personal exercise program, and communication (eg, chat, email) with either health care providers or patients. Limited information was found on barriers, facilitators, and users’ experiences. Methodological quality varied, with 13 studies being of moderate quality. The reported Web-based intervention elements appeared to be highly relevant to address the specific needs of cancer survivors as indicated by the IOM.

**Conclusions:**

We identified 7 common elements of interactive, Web-based interventions in chronic disease settings that could possibly be translated into eHealth recommendations for cancer survivors. While further work is needed to determine optimal intervention characteristics, the work performed in other chronic disease settings provides a basis for the design of an interactive eHealth approach to improve patient empowerment and physical activity in cancer survivors. This may subsequently improve their health status and quality of life and reduce their need for supportive care.

## Introduction

Due to improvements in cancer screening and treatment, the number of people living with cancer or that have been successfully treated for cancer is increasing rapidly [1]. Those people are often referred to as cancer survivors, and in the Netherlands this population is expected to increase from 419,000 in 2009 to 660,000 in 2020 [2]. Cancer survivors are increasingly approached as individuals with a chronic disease, with either on-going or intermittent impact on their health status and quality of life. Therefore, many of them need supportive and rehabilitative services to alleviate side effects of treatment and to cope with psychosocial problems such as fear of disease recurrence or with physical health problems such as a painful arm after breast cancer surgery. Furthermore, these services can be used for health promotion [3,4]. To minimize the time and costs involved with the need for such supportive care services in response to the raising number of cancer survivors, it may be useful to enhance patient empowerment.

Patient empowerment can contribute to control over patients’ health and health behavior. It is frequently described as having knowledge about one’s health, and being able and motivated to influence one’s health [5]. It refers to well-informed patients taking responsibility for their own health, to as great an extent as possible, and the expected benefits of improved quality of life [6]. It is expected that increasing patient empowerment will result in a reduced need for support from the health care system, thus lowering health care costs [7,8].

Another factor that positively contributes to quality of life is physical activity. A number of studies have demonstrated many beneficial effects of physical activity on physical and psychosocial well-being, both during and after cancer treatment [9-11]. This suggests that empowering cancer survivors and enabling them to become or stay physically active is very likely to be beneficial for both the patients and the society.

A promising medium for facilitating patient empowerment and physical activity is the Internet. Easily accessible, up-to-date, and tailored information can be provided, often in an interactive way. For example, patients could be asked to provide information or pose questions via a questionnaire to trigger either standardized or tailored feedback from the health care system (given automatically or by a health care provider). The Internet is increasingly used for the delivery of these interactive interventions, both for healthy individuals [12] and those with chronic conditions [13]. For cancer survivors, other eHealth initiatives do exist, such as online support groups, online patient education programs [14,15], informative tools for decision support, and various mobile apps that could be used independent of provider activities. However, there are very few interactive websites that aim to empower cancer survivors, especially in the area of physical activity. Previously, researchers have reviewed Web-based interventions that aimed to increase either patient empowerment or physical activity level, with promising results [13,16]. These reviews included studies that were focused primarily on healthy individuals (in some cases sedentary or overweight, [16]) or at increasing patient empowerment, but not physical activity levels, of individuals with chronic diseases [13]. In view of the increasing number of cancer survivors and the potential role that interactive Web-based interventions could play in stimulating empowerment and physical activity, it is important to learn from empirical evidence about the efficacy of such interventions in chronic diseases. Considering the comparable chronic nature of these diseases and cancer survivorship, it is plausible that interventions that contribute to managing chronic diseases other than cancer contain elements that are appropriate for cancer survivors as well.

This systematic review has 5 aims: (1) to describe the characteristics (content, length, frequency, duration) of interactive, Web-based interventions in diabetes, chronic obstructive pulmonary disease (COPD), (congestive) heart failure, cardiovascular disease, and cancer, (2) to summarize the effects of these interventions on patient empowerment and physical activity. (3) to identify barriers for and facilitators of the use of Web-based interventions and to describe users’ experiences with such websites, (4) to assess the methodological quality of the studies reviewed, and (5) to evaluate the possible relevance of these interventions for cancer survivors.

## Methods

### Search Strategy

We searched the literature in PubMed, Embase, and Scopus. The main search strategy combined four concepts: patient empowerment, physical activity, information technology (IT), and type of chronic disease. For each concept, several search terms were used (see Multimedia Appendix 1). Because we also wanted to identify IT that focussed on either physical activity or patient empowerment, we also searched PubMed for the combination of patient empowerment, IT, and type of chronic disease, and separately for the combination of physical activity, IT, and type of chronic disease. As these searches resulted in many duplicates, this dual search strategy was not repeated in Embase or Scopus. To retrieve other relevant publications, we examined the reference lists of the selected publications and reviews that were excluded based on eligibility criteria.

### Eligibility Criteria

We used the following inclusion criteria: (1) peer reviewed studies in English describing a randomized controlled trial (RCT), published between 1990 and November 20, 2012, (2) participants were adults and suffered from at least one of the following chronic diseases—cancer, diabetes, heart failure, cardiovascular disease, or COPD, (3) the intervention was Web-based and interactive, (4) the intervention group was compared to a similar patient group (receiving another intervention or usual care), and (5) the study included at least one outcome measure assessing patient empowerment and/or physical activity. For patient empowerment, relevant, related outcomes included self-efficacy, self-management, self-care behavior, and self-control. For physical activity, relevant outcomes could be based on self-report (eg, by questionnaire or interview), performance tests, or observation (eg, accelerometer data).

### Selection Method

The first author applied the eligibility criteria to the titles and abstracts. When the abstract was considered relevant or in case of ambiguity, two authors reviewed the full publication independently. In cases of disagreement, consensus was sought through discussion. When disagreement persisted, the judgement of a third reviewer was decisive.

### Data Extraction

The following information was extracted from each publication: study characteristics (source and year of publication, country of origin, aim, and sample size), patient characteristics (type of disease, age, gender, comorbidities, computer experience, and Internet use), intervention characteristics (content, duration, frequency, compliance, and dropout rate), outcome measures (instruments used, and effects on patient empowerment and physical activity), information about barriers to and facilitators of intervention use, and users’ reported experiences with the intervention. The first author independently extracted the data, and the second author checked the data extraction for 20% of the studies to determine inter-rater reliability. This was established by calculating the percentage of agreement. Consensus was reached by discussion. Due to the diversity of outcome measures, sample size, and intervention characteristics, it was not possible to conduct a formal meta-analysis.

### Quality Assessment

The methodological quality of the studies was evaluated, but did not serve as an eligibility criterion. We used a list that was an adapted version of the Cochrane Collaboration Back Review Group [17], which was used previously in a systematic review of Internet-based physical activity interventions by van den Berg and co-workers. These authors modified the Cochrane list to better suit the type of studies they examined. For example, the Cochrane list contained the item “description of and acceptable dropout rate”, which was changed into “description of dropout rate plus comparison of dropouts with completers”. In addition, they deleted some items because they were not relevant for Web-based interventions [16]. For our review, one additional change was made. “Long-term follow-up measurement” was defined as an outcome assessment more than 3 months after the post-intervention measurement. The final list of criteria included 13 items relating to the selection of patients, the intervention, outcome measurements, and statistics. The complete list can be found in Table 3, in which the outcomes of the methodological quality assessment are shown.

For each study, all criteria were scored with yes, no, or unclear, resulting in a maximum quality score of 13. In line with other researchers [16], we considered studies obtaining at least two-thirds of the total score (ie, ≥9 points) to be of high quality. Studies scoring 5 to 8 points were rated as moderate quality, and studies scoring lower than 5 points were rated as low quality. Quality assessment was performed by the first author, while the second author assessed the quality of a random sample of 4 studies. The inter-rater reliability was calculated as percentage of agreement on 52 aspects (4x13 criteria). Disagreements between researchers were discussed to reach consensus.

### Evaluation of Potential Relevance for Cancer Survivors

To evaluate the relevance of the selected interventions for cancer survivors, we used the 5 factors included as characteristics of cancer survivorship identified by the Institute of Medicine (IOM): surveillance, management of late effects, rehabilitation, psychosocial support, and health promotion [4]. We evaluated whether the interventions reviewed could be mapped onto these 5 features of cancer survivorship care.

### Selection of Publications

**Figure 1:**
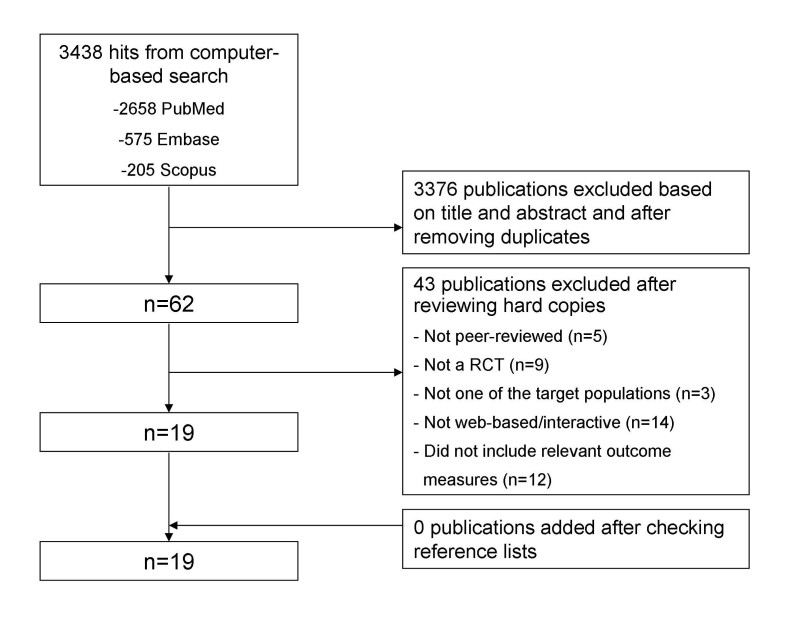
Flow chart of the search process.

The initial search yielded 3438 hits. Based on titles and abstracts, 62 publications were selected. The full text of these 62 publications were reviewed, resulting in a selection of 19 publications that met all eligibility criteria [18-36]. A review of the reference lists of these publications, as well as the reference lists of the excluded reviews did not result in any additional studies. See Figure 1 for a flow chart of the selection process. For the cancer setting, we found 46 papers that met several of our inclusion criteria, but not all. Reasons for exclusion were diverse, varying from not being an RCT (eg, design papers or non-randomized pilot studies) to inappropriate outcome measures or not being Web-based and/or interactive (eg, a CD-ROM).

### Data Extraction

Reviewers’ ratings were in agreement for 89.5% (68/76) of the data extraction elements from the sample. This can be considered as a high level of agreement according to the guidelines of Landis and Koch [37], and justified the decision to have only one of the authors carry out the data extraction for the remaining studies.

### Study Characteristics

All papers were published in year 2000 or later, with most being published after 2005. The 19 publications described 18 unique studies; two papers by Glasgow et al described the same study at different assessment time-points (4 and 12 months, [21,22]). Twelve studies were conducted in the United States. The remaining studies took place in Canada (n=2), Korea (n=1), Norway (n=2), and Australia (together with the United States, n=1). Sample sizes at baseline varied between 15 and 1665. In the majority of RCTs, one group was exposed to a Web-based intervention and was compared to control group with usual care (n=7), an information only condition (n=2), an observational control group (n=1), a face-to-face intervention (n=1), both a print material intervention and usual care (n=1), or both a face-to-face intervention and an information only condition (n=1). In two RCTs, two intervention groups were compared with either control group with usual care or enhanced usual care. In one RCT, two groups receiving a Web-based intervention were compared with an information only condition. Finally, in two RCTs, two intervention groups were compared. Those groups received the same basic intervention, but with a different focus (high vs low self-efficacy and lifestyle goals versus structured goals).

### Patient Characteristics

Studies included patients with diabetes (n=11), heart failure (n=3), COPD (n=1), cardiovascular disease (n=1), cancer (n=1), and mixed patient groups (heart disease, lung disease, type 2 diabetes; n=1). The overall mean age of the participants was 60 years (SD 8.5 years, range 40-76 years). For the 18 studies that reported on gender, the median percentage of women was 53.1% (range 6.0%-73.3%). Individuals with comorbid conditions were excluded in 7 studies, and 6 studies provided information about comorbidity (eg, mean number of comorbid conditions). Only 6 studies collected information on participants’ prior experience with computers and/or Internet use. Both computer experience and Internet use were assessed with a variety of self-reported questionnaire items, ranging from times per week to years of experience, making it difficult to compare across studies.

### Intervention Characteristics

Intervention characteristics for both intervention and control groups are described in Table 1. The degree of detail provided about the interventions varied greatly across studies. There was large variation in the duration, frequency, and content of the interventions. Duration varied between 1 month and 1 year (mean 23 weeks, SD 19 weeks). The intended frequency or intensity of the interventions was not clearly described in the majority of the papers. In some papers, a schedule for intervention use was proposed [23,25,36], whereas in other papers only information about actual use was reported (eg, number of logins, percentage of individuals that used the different intervention elements).

Although the content of the interventions differed, 7 key elements used in the majority of the interventions were identified, including interventions that had significant effects and those that did not. These elements were used in different combinations and were adapted to the specific patient population. The first element was *education*, which included information about various aspects of the chronic health condition such as medication, nutrition, exercise, coping, and symptom management, provided via electronic newsletters, pamphlets, slides, or a digital library with articles. The second element, *self-monitoring*, involved uploading or registration of data such as blood glucose levels, blood pressure, medication use, food intake, and exercise behavior. In the study of Nguyen and colleagues for example, individuals had to submit real-time information about dyspnea, cough, and sputum via their computer or smart phone [29]. This was often followed by the third element, *feedback/tailored information*. Based on the uploaded data, patients received individual feedback, which included individually tailored exercise advice, or a graphical overview of blood glucose levels for example. Other forms of tailored information included access to the medical record, medication reminders, and tips for overcoming personal exercise barriers. Fourth, interventions could include *self-management training*, involving lessons about the management of symptoms, psychosocial aspects, and fatigue. An important aspect of self-management training was goal setting. The fifth element, found only in interventions involving physical activity, was a *personalized exercise program* that was adapted on the basis of self-reported and/or objective physical activity data obtained before and during the intervention. The exercise programs took the individual's needs, preferences, and possibilities into account. In the study of McKay and co-workers for example, patients went through a 5-step process to select their personal motivators, goals, preferred activities and schedule, and to identify their personal barriers [27]. Elements 6 and 7 both involved *communication, either with health care providers or with fellow patients*, respectively, using communication routes like email, live chat, videoconferences, and discussion boards (forums). Communication with health care providers was often used for questions, encouragement, and emotional support, whereas communication with fellow patients was included to share experiences, exchange information, and provide support. Additionally, elements that were used in only some studies were an educational quiz and periodic reminders for website use.

The overall percentage of dropouts varied between 0.0% and 52.3% (median 17.5%). For the intervention groups (including the control groups that also received a Web-based program) the median dropout rate was 19.7% and for the control groups this was 14.0%. Compliance with the intended intervention varied between 36.6% and 96.0% for the 9 studies that reported on it. The remaining studies did not report compliance, but described aspects of website use, such as number of logins, percentage of people using a certain feature, minutes per session, or percentage of tasks completed. Intervention use varied greatly between studies and participants. All studies that monitored website use found a decline during the intervention period. There was no obvious relationship between dropout rates, compliance, and website use on the one hand, and patient and intervention characteristics on the other hand.

### Outcome Measures

Table 2 presents patient empowerment and physical activity outcomes and dropout rates. A range of outcome measures was used (eg, different self-efficacy scales, diverse measures of different forms of physical activity).

A total of 13 studies included one or more patient empowerment measures. In 4 studies, patient empowerment increased significantly (*P*<.05) in the intervention group compared to usual care or observation [19,25,34,36], while in 3 studies this increase was reported for both groups [18,22,28] (ie, both the Web-based intervention group and the comparison group improved irrespective of receiving a Web-based, a face-to-face intervention or usual care). In the study of Nguyen et al for example, both the individuals receiving the Web-based intervention and those having personal contact improved on a measure of self-efficacy [28]. Two studies yielded mixed results, with 1 of 2 outcome measures showing a significant increase [24,35]. For example, Wangberg et al measured both self-efficacy and self-care behavior, but observed improvement only in the latter. The remaining 4 studies did not observe a significant change in patient empowerment [26,29,31,32] for either the intervention group or the usual care group.

Of the14 studies that assessed physical activity, 2 reported significant improvement (*P*<.05) for the intervention group compared to usual care [33,36] (eg, an increase in the number of individuals who exercised regularly or in physical activity behavior). Increases in physical activity were found for both groups in 6 studies [21-23,27,28,30]. For instance, McKay et al compared their Web-based group with an information only approach. Both groups improved their moderate/vigorous exercise behavior as well as their walking performance. Three studies [24,26,29] found mixed results, with one of their outcome measures being non-significant and the others showing a significant increase. For example, Lorig et al found no change in aerobic exercise behavior but did observe an increase in stretch/strength exercise [26]. Finally, 3 studies did not find any effects on physical activity [18,20,25].

**Table 1:** Intervention characteristics.

Study	Patient group (sample size)	Study design	Intervention	Follow-up period
Artinian et al (2007)	Congestive heart failure (n=18)	Pilot RCT with an intervention group and a comparison group receiving usual care	Home care monitoring system: - pamphlet with education about self-care behavior - medication reminders - questions & response - registration of pill taking	3 months
Bond et al (2010)	Diabetes (n=62)	RCT with an intervention group and a comparison group receiving usual care	Focus on self-management and psychosocial well-being: - usual care - instructions (about issues regarding disease management) - interaction with study nurse - uploading data & receiving feedback - online educational discussion group - peer support via email and instant messaging	6 months
Glasgow et al (2003)	Type 2 diabetes (n=320)	RCT with 3 intervention groups and an Internet information only comparison group (library with articles, automated dietary goal setting, online assessments)	Aspects of information only and: (1) Tailored self-management training: - online professional suggesting tailored strategies - question and answer with dietician - blood glucose upload and dietary databases plus graphical feedback	10 months
			(2) Peer support: - exchange of information, coping strategies, and emotional support on a forum - live chat - 5 electronic newsletters	
			(3) both 1 and 2	
Glasgow et al (2010, 2011)	Type 2 diabetes (n=463)	RCT with 2 intervention groups and an enhanced usual care comparison group (health risk appraisal feedback, recommendations of preventive care behavior)	Self-management program with: (1) Minimal support: - goal selection - progress recording - feedback - community resources - quiz questions - motivational tips - periodic prompting	4 months and 12 months, respectively
			(2) Moderate support: - aspects of minimal support - follow-up calls - invitation for a group visit with other participants	
Kim & Kang (2006)	Type 2 diabetes (n=73)	RCT with an intervention group, a print-material comparison group (booklets with tailored exercise strategies), and a comparison group receiving usual care	Physical activity (PA) intervention: - general information - assessment tools for physical and psychological readiness for exercise - stage-based individual information about goal setting and exercise planning - question and answer board - interactive and animated features - exercise test (in the lab), followed by an individualized physical activity prescription	12 weeks
Liebreich et al (2009)	Type 2 diabetes (n=49)	RCT with an intervention group and a comparison group receiving usual care	Website and counselling: - link to clinical practice guidelines for physical activity - interactive features (physical activity logbook, forum, email counselling) - education/tips - weekly topic (eg, goal setting, time management)	12 weeks
Lorig et al (2006)	Heart & lung disease, type 2 diabetes (n=958)	RCT with an intervention group and a comparison group receiving usual care	Self-management program and usual care: - individual exercise program - management of symptoms, fatigue, emotions, problems - motivational email reminders - overview of medications - interaction with moderator - action planning - feedback	12 months
Lorig et al (2010)	Type 2 diabetes (n=761)	RCT with 2 intervention groups (only difference was email support; analyzed together) and a comparison group receiving usual care	Self-management program: - 6 weekly sessions with different topics - bulletin board - exercise logs and monitoring tools - communication with facilitators	18 months
McKay et al (2001)	Type 2 diabetes (n=78)	RCT with an intervention group and an information only comparison group (library articles, glucose tracking plus feedback)	PA intervention: - feedback on baseline activity levels - personalized PA program and PA database - personal coach counselling and support - communication with other intervention participants	8 weeks
Nguyen et al (2008)	COPD (n=50)	RCT with an intervention group and a face-to-face intervention comparison group (same intervention components)	Self-management program: - education and skills training - tailored exercise planning - self-monitoring of symptoms and exercise - personalized feedback	6 months
Nguyen et al (2012)	COPD (n=125)	RCT with an intervention group, a face-to-face intervention group and a general health education comparison group (home visit, monthly face-to-face education sessions, phone calls with health information)	Self-management program (same components for online and face-to-face group): - dyspnea and exercise consultation at home (once) - individualized exercise plan - self-monitoring and reinforcement - education - skills training - peer interactions	12 months
Richardson et al (2007)	Type 2 diabetes (n=35)	Pilot RCT with 2 intervention groups (with a focus on either lifestyle goals or structured goals)	Pedometer-based walking program with a focus on: (1) Lifestyle goals (targeting accumulated steps) - access to a personally-tailored Stepping Up to Health Web page - tailored motivational messages - tips about managing diabetes - automatically calculated goals (based on pedometer results) - feedback about performance toward goals	6 weeks
			(2) Structured goals (only targeting steps taken during bouts of at least 10 minutes with at least 60 steps per minute): - intervention see (1)	
Ross et al (2004)	Congestive heart failure (n=107)	RCT with an intervention group and a comparison group receiving usual care	Secure Web-interface to 3 features, and reminders for system use: - medical record - educational guide - messaging system	12 months
Ruland et al (2012)	Breast and prostate cancer (n=325)	RCT with an intervention group and an information only comparison group	Self-management program: - self-monitoring plus tailored self-management support - information - communication with other patients and expert nurses - diary for personal notes	12 months
Tomita et al (2009)	Heart failure (n=40)	RCT with an intervention group and a comparison group receiving usual care	Self-management program in addition to usual care: - informational support - recording vital signs and exercise - appraisal support (feedback) - emotional support	12 months
Trief et al (2007)	Diabetes (n=1665)	RCT with an intervention group and a comparison group receiving usual care	Telemedicine case management: - access to educational materials - upload data on blood glucose and blood pressure readings - videoconference with a nurse case manager and dietician (to educate patients, facilitate goal setting/self-management, and discuss concerns)	12 months
Wangberg (2008)	Type 2 diabetes (n=60)	RCT with 2 intervention groups (with a focus on either high or low self-efficacy)	Self-care intervention tailored to either high or low self-efficacy: - behavior exercises (including monitoring and graphic feedback) - information - quizzes with feedback - videos of peers - videos of lectures from health personnel	1 month
Zutz et al (2007)	Cardiovascular disease (n=15)	Pilot RCT with an intervention group and an observational control comparison group (no contact with either the research staff or the hospital)	Cardiac rehabilitation program: - chat sessions with health care professionals - education sessions (slides) - monitoring of blood and exercise - group chat sessions	12 weeks

**Table 2:** Intervention outcomes and dropout rates.

Study	Patient empowerment outcome measure^c^	Patient empowerment outcomes^a,c^	Physical activity outcome measure^c^	Physical activity outcomes^a,c^	Dropout rate (overall)
Artinian et al (2007)	Revised Heart Failure Self-Care Behavior Scale	Self-care + (*P*=.02)^e^	6 Minutes Walking Test (6MWT)	Exercise performance - (*P*=.42)	0.0%
Bond et al (2010)	Diabetes Empowerment Scale	Self-efficacy + (*P*<.05)^b,e^	X	X	0.0%
Glasgow et al (2003)	X	X	Physical Activity Scale for the Elderly	Physical activity - (*P=*.41)	18.0%
Glasgow et al (2010) Glasgow et al (2011)	Diabetes Self-Efficacy scale	Self-efficacy 4 months X	Community Health Activities Model Program for Seniors Questionnaire	Caloric expenditure in physical activity 4 months: + (*P=*.04)^d^	4 months 17.5%
		12 months + (*P*<.10)^b, f^		12 months: + (*P*<.05)^b,d^	12 months 22.7%
Kim & Kang (2006)	X	X	Self-report instrument adapted from the 7-day physical activity questionnaire (frequency, duration, intensity)	Metabolic equivalents (MET) x hours/week + (*P*<.001)^d^	0.0%
Liebreich et al (2009)	Likert scale (1-5); 12 items	Self-efficacy - (*P=*.31)	Godin Leisure-Time Exercise Questionnaire (GLTEQ)	MET minutes + (*P*=.04)^d^	10.3%
	Likert scale (1-5); 4 items	Behaviour capacity + (*P*=.001)^d^	GLTEQ	Unweighted minutes + (*P*=.01)^d^	
			Incorporated in GLTEQ (times/week, average time per session)	Resistance training - (*P=*.06)	
Lorig et al (2006)	Likert scale (1-10)	Self-efficacy - (*P*=.06)	Scale (0-4) measuring minutes of exercise per week	Stretch/strength exercise + (*P*=.02)^d^	18.8%
				Aerobic exercise - (*P*=.70)	
Lorig et al (2010)	Patient Activation Measure	Patient activation + (*P*=.01)^d^	A physical activities scale (minutes/week)	Aerobic exercise - (*P*>.05)^b^	15.8%
	Diabetes Self-Efficacy scale	Self-efficacy + (*P=*.02)^d^			
McKay et al (2001)	X	X	Behavioural Risk Factor Surveillance System	Moderate/vigorous exercise + (*P*<.001)^e^	12.7%
				Walking + (*P*<.001)^e^	
Nguyen et al (2008)	Single question on a 0- to 10- point response scale	Self-efficacy + (*P*=.02)^e^	Self-report (frequency & duration)	Endurance exercise + (*P*=.001)^f^	24.0%
				Strength exercise + (*P*<.001)^f^	
			List of 5 descriptions	Stages of change + (*P*=.05)^f^	
			6MWT	Exercise performance + (*P*=.05)^d^	
Nguyen et al (2012)	Single question on a 0- to 10- point response scale	Self-efficacy - (**P*=.06)*	6MWT	Exercise performance + (**P*<.001)* ^*e*^	12.0%
			Incremental treadmill test	Exercise performance - (**P*>.05*)^b^	
			Lifting a wooden dowel	Arm endurance + (**P*=.04*)^f^	
			Self-report (frequency & duration)	Endurance duration + (**P*=.04*)^f^	
				Endurance frequency + (**P*=.001*)^e^	
				Strengthening frequency + (**P*<.001*)^f^	
Richardson et al (2007)	X	X	Pedometer (Omron HJ-720IT)	Total steps + (*P*=.003)^d^	14.0%
				Bout steps + (*P*<.001)^d^	
Ross et al (2004)	Kansas City Cardiomyopathy Questionnaire (self-efficacy domain)	Self-efficacy - (*P=*0.08)	X	X	24.0%
Ruland et al (2012)	Cancer Behavior Inventory version 2.0	Self-efficacy - (**P*=.26)*	X	X	24.6%
Tomita et al (2009)	X	X	Self-reported frequency of exercise; participants exercising 2-3 times/week or more were seen as exercisers	Number of exercisers + ( *P=*.001)^d^	19.8%
Trief et al (2007)	Diabetes Self-Efficacy scale	Self-efficacy + (*P*<.001)^d^	X	X	52.3%
Wangberg (2008)	Perceived Competence Scales	Self-efficacy - (*P=*.17)	X	X	45.9%
	Summary of Diabetes Self-Care Activities measure	Self-care behavior + (*P*=.026)^d^			
Zutz et al (2007)	Likert scoring	Self-efficacy (exercise- specific) + (*P*<.05)^b,d^	Minnesota Leisure Time Physical Activity Questionnaire	Physical activity + (*P*<.05)^b,d^	6.7%
			Symptom-limited treadmill exercise stress test	Exercise capacity + (*P*<.05)^b,d^	

^a^+ is a positive effect; - is no effect

^b^The researchers did not provide specific *P* values.

^c^X=not applicable

^d^Positive effect for the Web-based intervention group(s) only

^e^Positive effect for all groups (including usual care)

^f^Positive effect for the Web-based intervention group(s) and other intervention groups

### Barriers for and Facilitators of Intervention Use and Reported Users’ Experiences

Five studies reported on perceived barriers, whereas no studies reported on perceived facilitators of the use of interactive, Web-based interventions. Perceived barriers were typically of a technical nature, including problems with Internet connection, slow loading of website, security concerns, discomfort with using the computer or Internet, and problems with related hardware (eg, PDA, monitor). Ten studies described some users’ experiences, for example, satisfaction scores and a judgment of intervention content. In general, patient satisfaction was high. The personalized nature of the interventions was often cited by participants as being important. In one study [31], nurses and physicians reported that their workload did not increase as a result of the intervention.

### Methodological Quality

The concordance between reviewers in rating the methodological quality for the sample of papers evaluated was high (90.3% (47/52), [37]). For this reason, the first researcher independently performed the quality assessment for the remainder of the studies. The two papers of Glasgow that described the same study were judged together for methodological quality. Table 3 shows that 3 of 18 studies [21,22,28,29] obtained a score of 9 or higher, indicating good methodological quality. Two studies were of low quality [24,31] and the remaining studies were of moderate quality, with most studies scoring a 5 or 6. All studies specified eligibility criteria and employed a comparable timing of outcome assessment for the different groups. The majority of the studies reported dropout rates, including a comparison between completers and non-completers, and gave point estimates together with measures of variability. Only a minority of studies (maximum n=7) provided information about the method of randomization, described their intervention explicitly, performed a power calculation and used an intention-to-treat approach to the data analysis. Most studies were unclear about concealing treatment allocation and blinding of the outcome assessor. In one study, groups were not similar at baseline, and for one study this was not clear. Only 2 studies described a long-term follow-up measurement. Because 12 out of 18 studies were of moderate quality, it was not possible to determine whether differences in outcomes were related to methodological quality.

**Table 3:** Methodological quality assessment.^a,b^

	1	2	3	4	5	6	7	8	9	10	11	12	13	Total score^d^
Artinian et al (2007)	√	X	?	√	X	√	?	√^c^	X	√	X	X	√	6
Bond et al (2010)	√	X	?	√	X	X	√	√^c^	X	√	√	X	√	7
Glasgow et al (2003)	√	X	?	√	X	X	?	√	X	√	√	X	√	5
Glasgow et al (2010, 2011)	√	√	?	√	√	√	?	√	X	√	√	√	√	10
Kim & Kang (2006)	√	X	?	√	X	X	?	√^c^	X	√	X	X	√	5
Liebreich et al (2009)	√	X	?	X	X	√	?	X	X	√	X	X	√	4
Lorig et al (2006)	√	X	?	√	√	X	?	√	√	√	X	√	√	8
Lorig et al (2010)	√	√	?	√	X	X	?	√	√	√	X	√	√	8
McKay et al (2001)	√	√	?	√	X	X	?	√	X	√	X	X	√	6
Nguyen et al (2008)	√	√	√	√	√	√	√	√	X	√	X	√	√	11
Nguyen et al (2012)	√	X	X	√	√	√	√	√	X	√	√	√	√	10
Richardson et al (2007)	√	X	?	?	√	√	?	X	X	√	X	X	√	5
Ross et al (2004)	√	√	?	√	X	X	?	X	X	√	X	X	X	4
Ruland et al (2012)	√	√	?	√	X	√	?	X	X	√	√	√	√	8
Tomita et al (2009)	√	X	?	√	√	√	?	X	X	√	√	√	X	7
Trief et al (2007)	√	X	?	√	X	X	√	√	X	√	X	√	X	6
Wangberg (2008)	√	X	?	√	X	X	?	√	X	√	√	X	√	6
Zutz et al (2007)	√	X	?	√	√	√	?	X	X	√	X	X	√	6

^a^1=specification of eligibility criteria; 2=method of randomization explained; 3=treatment allocation concealed; 4=groups similar at baseline; 5=explicit description of interventions; 6=description of compliance; 7=outcome assessor blinded; 8=description of dropout and comparison with completers; 9=long-term follow-up (> 3 months after post-intervention assessment); 10=timing of outcome assessment comparable; 11=sample size described with power calculation; 12=intention-to-treat analysis; 13=point estimates and measures of variability

^b^√=reported item; X=unreported item; ?=unclear item

^c^Dropout rate was 0%

^d^Maximum score was 13

### Evaluation of Potential Relevance for Cancer Survivors

Our judgement of the relevance of the intervention elements for the cancer survivorship setting was based on their *Web-based* application (as opposed to their usefulness, in general). Table 4 (the more cancer-related recommendations) and Table 5 (the more health-related recommendations) show how these intervention elements could be mapped onto the recommendations for survivorship care as described by the IOM [4]. Five intervention elements contributed to all recommendations, and two elements (personal exercise program and communication with fellow patients) would only be inappropriate for long-term follow-up/surveillance. The specific content of each element when adapted to the oncology setting depended on the recommendation for which it was used. For example, information provision will differ for surveillance versus healthy lifestyle recommendations. Similarly, a personal exercise plan for rehabilitation after surgery differs from an exercise plan that aims to enhance general physical activity levels.

**Table 4:** Proposed application of intervention elements that could enhance cancer survivorship care based on findings from this review (cancer-related recommendations).

	Recommendations for survivorship care	
Elements of Web-based intervention	Long-term follow-up/surveillance	Management of (late) effects
Education	Information about reasons for surveillance	Information about possible late effects of cancer treatment
	Recommendations for self-screening	
Self-monitoring	Reporting results of self-screening	Upload of relevant vital signs (eg, pain scores, blood values)
Feedback/Tailored information	A personal follow-up schedule with frequency and type of screening	Advice for managing (late) effects as identified by self-monitoring data
	Feedback on reported self-screening	
Self-management training	Training aimed at performing regular self-screening	Training to learn to cope with late effects of cancer treatment
Personal exercise program	X	Individual exercise advice to prevent or reduce (late) effects, taking into account a survivor's specific needs and preferences
Communication with health care provider	Possibility to ask questions about follow-up and self-screening	Possibility to ask questions about symptoms and how to deal with them
Communication with fellow patients	X	Share experiences and tips about managing (late) effects

^a^X=application not relevant

**Table 5:** Proposed application of intervention elements that could enhance cancer survivorship care based on findings from this review (health-related recommendations).

	Recommendations for survivorship care		
Elements of Web-based intervention	Rehabilitation	Psychosocial support	Health promotion
Education	Information about the importance of and possibilities for rehabilitation	Information about possible psychosocial problems and possibilities to solve them	Information about the importance of and ways to obtain a healthy lifestyle (eg, physical activity, nutrition, smoking cessation)
Self-monitoring	Upload of relevant vital signs (eg, blood pressure, lung function) or exercise behavior (either self-reported or objective)	Questionnaire(s) measuring psychosocial aspects	Upload of relevant data such as food intake and exercise behavior
Feedback/Tailored information	Rehabilitation advice based on self-monitoring data	Advice for dealing with psychosocial problems as identified with questionnaires; following the stepped care principle	Health advice based on uploaded data; following the stepped care principle
Self-management training	Training to learn to sustain doing rehabilitation exercises	Training aimed at coping with psychosocial problems like anger, fear or frustration	Training aimed at obtaining and sustaining a healthy lifestyle
Personal exercise program	Individual exercise advice aimed at rehabilitation, taking into account a survivor's specific needs and preferences	Individual exercise advice, taking into account a survivor's specific needs and preferences	Individual exercise advice, taking into account a survivor's specific needs and preferences
Communication with health care provider	Possibility to ask questions about rehabilitation	Possibility to ask questions about psychosocial problems; receiving support	Possibility to ask questions about exercise advice
Communication with fellow patients	Share experiences and tips about rehabilitation	Share experiences and tips about dealing with psychosocial problems	Share experiences and tips about health behavior
		Provide support	Provide support

## Discussion

### Principal Findings

In this paper we have systematically reviewed the empirical literature on Web-based interventions for people with diabetes, COPD, heart failure, cardiovascular disease, and cancer, and have evaluated their potential relevance for cancer survivors. Nineteen publications covering 18 unique studies were included in this review. The RCTs varied greatly in content, duration, and frequency. Significant, positive effects on patient empowerment were found in 4 studies and 2 studies reported positive effects on physical activity. The remaining studies reported mixed results or no significant differences between intervention and comparison groups (ie, either both groups or neither group improved) on these outcomes. The information we could obtain about barriers and facilitators for intervention use and users’ experiences was limited. Nevertheless, we identified 7 elements that were common for the majority of interventions: education, self-monitoring, feedback/tailored information, self-management training, personal exercise program, and communication (with either health care providers or fellow patients). We were able to map these elements onto eHealth features for the recommendations for survivorship care of the IOM.

The 7 common intervention elements were used in different combinations and were adapted to the specific patient population. It is therefore not possible to make a judgment about the individual contribution of these elements to intervention outcomes. Future studies should be more structured, in order to determine the role of individual intervention elements and should also take the duration and frequency of interventions into account. In most studies no intervention schedule was prescribed. Rather, the intensity, frequency, and duration of website use were determined by the participants themselves. In contrast, structured rehabilitation programs usually have schedules to which patients are expected to adhere (eg, performing moderate intensity physical activity (running or cycling) for 30 minutes, 3 times a week, during a 12-week period). It is debatable whether Web-based interventions should or should not have a structured program, but it is conceivable that a certain combination of duration and frequency is optimal for achieving improved patient empowerment and physical activity. A recent review of Web-based interventions for type 2 diabetes [38] indicated that interventions of longer duration (more than 12 weeks) resulted in better outcomes, and it is likely that the same is valid for cancer survivors. However, future studies need to confirm this.

The relative importance and value of intervention elements, duration, and frequency on outcomes is not yet clear. Other factors may also have played a role in the large variation in patient empowerment and physical activity outcomes observed in the studies reviewed. These include the different measurement tools that were used within and between studies, different sample sizes and different periods between the start of the intervention and the post-intervention measurement. To facilitate future meta-analyses, new investigations should preferably use uniform outcome measures and time intervals for the outcome assessment. The need for a uniform measure of patient empowerment was also pointed out in a paper that discussed the role of assessing patient empowerment in health care evaluation [39].

Another issue to be considered is that, in the majority of studies where no significant differences between groups were observed, significant, positive effects were found for all groups. In many of these studies, the comparison group(s) received an intervention as well. This may have limited the possibility of detecting an effect in favour of the Web-based interventions. More generally, it is becoming increasingly difficult to establish appropriate control groups, because the usual care situation is evolving rapidly. Although previous studies have shown that effects on knowledge and behavior change were higher for individuals using a Web-based intervention than for individuals using a non-Web-based intervention [40], more work is needed to determine whether this also applies to cancer survivors.

It would have been useful if the RCTs reviewed had provided more information on barriers and facilitators for intervention use. Insight into these factors is very important, because Web-based interventions are often characterised by high dropout rates [41]. Dropout can refer to patients being lost to follow-up or to patients not using the intervention. Bennett and Glasgow indicated that an important reason for dropout is loss of interest [42]. Furthermore, 2 literature reviews showed that peer support, counsellor support, email and phone contact, frequent website updates, record keeping, and individualized feedback were related to sustained intervention use (and conversely, to less dropout, [43,44]). Most of these components were present in the studies included in the current review. The mean percentage of dropouts in the Web-based intervention groups of the studies reviewed was 19.7%, which is comparable to the dropout rate found in another review (21.0%, [40]). More research on program adherence is needed. Or, in other words, it should be determined “what works and why” [44].

The assessment of the methodological quality of the studies reviewed suggests a number of areas in which there is room for improvement. Future RCTs in the field of Web-based interventions could be improved by clearly describing the method of randomization, concealment of treatment allocation, and an adequate description of sample size calculation. Additionally, researchers should preferably describe explicitly their intervention(s), including specific information about intervention elements, length, frequency, and duration. Studies should carry out the statistical analysis on an intention-to-treat basis (as opposed to only analyzing the participants who completed the intervention). This is important because participants who complete an intervention may differ from those who do not, as a result of which intervention effects may be over- or underestimated. Because it is often the goal to not only enhance patient empowerment and facilitate a physically active lifestyle in the short-term, but to sustain these outcomes over a longer period of time, it is important that RCTs include not only immediate post-intervention outcome assessment, but also longer-term follow-up assessments.

Web-based interventions are being developed at a rapid pace. This is also true for Web-based interventions for cancer survivors. In this review we identified only 1 paper in the cancer field that met our eligibility criteria. Recently, however, positive results of a Web-based intervention to reduce depression in cancer survivors [45], and of a Web-based, tailored education program to reduce cancer-related fatigue and anxiety [46] have been reported. Several additional RCT’s of Web-based interventions for cancer patients and survivors are currently on-going [47]. It is likely that in several years there will be sufficient, mature studies to facilitate a formal meta-analysis to more precisely determine the effects of Web-based interventions for both chronic diseases and cancer, rather than the more qualitative review presented here.

Although we identified 7 elements of eHealth interventions that may be relevant for cancer survivors, based on the available evidence, we could not determine which of these elements are the most important and effective. It was also unclear which combinations of intervention elements would be optimal. However, the benefit of the educational element for cancer survivors was supported by a review, which showed that cancer survivors who received sufficient information reported a better quality of life [48]. An emerging approach in cancer survivorship that may encompass or incorporate many of the intervention elements described in the eHealth literature is the use of a survivorship care plan. Such a plan includes a summary of the individual patient’s diagnosis and treatment, as well as recommendations for appropriate follow-up care [4]. Currently, survivorship care plans are typically provided on paper, and consequently are quite static documents. There is no reason why they cannot be adapted for eHealth use, including interactive elements.

### Conclusion

In conclusion, our review suggests that Web-based, interactive interventions have a beneficial effect on patient empowerment and/or physical activity in people with various chronic conditions. Program elements that were frequently observed included education, self-monitoring, feedback/tailored information, self-management training, personal exercise program, and communication (with either health care providers or fellow patients). Although the results of these studies did not necessarily differ from those of traditional interventions, it is likely that the elements increased patient centeredness and efficiency of the interventions. Empowered individuals who are physically active are likely to have a better health status and quality of life, therefore the use of interactive Internet interventions in this field would appear promising. Further research is needed to establish optimal intervention characteristics and specific effects in cancer survivor populations. Future studies should also identify perceived barriers for and facilitators of the use of Web-based interventions. The studies that have been conducted in other chronic diseases are likely to constitute a basis for the development of an interactive, Web-based intervention to effectively empower the rapidly growing number cancer survivors.

## Multimedia Appendix 1

Search strategy in PubMed.

## Abbreviations

6MWT: 6 Minutes Walking Test
COPD: chronic obstructive pulmonary disease
GLTEQ: Godin Leisure-Time Exercise Questionnaire
IOM: Institute of Medicine
IT: information technology
MET: metabolic equivalents
PA: physical activity
RCT: randomized controlled trial
