# Supplementary material for: A Systematic Review of Web-Based Interventions for Patient Empowerment and Physical Activity in Chronic Diseases: Relevance for Cancer Survivors
Source: J Med Internet Res. 2013 Feb 20;15(2):e37. doi: 10.2196/jmir.2281 (PMC3636300; doi:10.2196/jmir.2281)
Supplement: Supplementary file 1 [file jmir_v15i2e37_app1.pdf]

#1	("patient empowerment" OR "empowerment" OR "self- efficacy" OR "mastery" OR "self-management" OR "self-control" OR "self-confidence" OR "perceived control" OR "perceived competence" OR "competence" OR "power" OR "self-determination" OR "enhanced control" OR "patient participation" OR "motivation" OR "locus of control")
#2	("physical exercise" OR "physical activity" OR "exercise" OR "physical training" OR "active lifestyle" OR "activities of daily living")
#3	("ict" OR "information and communication technology" OR "web-based" OR "internet" OR "information technology" OR "online" OR "computer assisted" OR "telemedicine" OR "telecommunication" OR "computer assisted instruction" OR "user-computer interface" OR "computerized")
#4	("cancer patient" OR "cancer survivor" OR "cancer survivorship" OR "cancer" OR "chronic obstructive pulmonary disease" OR "diabetes" OR "heart failure" OR "cardiovascular disease")
#5	#1 AND #2 AND #3 AND #4
#6	#1 AND #3 AND #4
#7	#2 AND #3 AND #4

Search strategies in Embase and Scopus were comparable, but did not include #6 and #7. Specific key term and and requirements for each database (with regard to brackets and commas) were taken into account.
